# Impact of ezetimibe on plasma lipoprotein(a) concentrations as monotherapy or in combination with statins: a systematic review and meta-analysis of randomized controlled trials

**DOI:** 10.1038/s41598-018-36204-7

**Published:** 2018-12-14

**Authors:** Amirhossein Sahebkar, Luis E. Simental-Mendía, Matteo Pirro, Maciej Banach, Gerald F. Watts, Cesare Sirotri, Khalid Al-Rasadi, Stephen L. Atkin

**Affiliations:** 1 Biotechnology Research Center, Pharmaceutical Technology Institute, Mashhad University of Medical Sciences, Mashhad, Iran; 2 Neurogenic Inflammation Research Center, Mashhad University of Medical Sciences, Mashhad, Iran; 3 School of Pharmacy, Mashhad University of Medical Sciences, Mashhad, Iran; 4 Biomedical Research Unit, Mexican Social Security Institute, Durango, Mexico; 5 Unit of Internal Medicine, Angiology and Arteriosclerosis Diseases, Department of Medicine, University of Perugia, Perugia, Italy; 6 Department of Hypertension, WAM University Hospital in Lodz, Medical University of Lodz, Zeromskiego 113, Lodz, Poland; 7 Polish Mother’s Memorial Hospital Research Institute (PMMHRI), Lodz, Poland; 8 School of Medicine, Faculty of Health and Medical Sciences, University of Western Australia, Perth, Australia; 9 Lipid Disorders Clinic, Cardiometabolic Services, Department of Cardiology, Royal Perth Hospital, GPO Box X2213 Perth, Australia; 10 Centro Dislipidemie, A.S.S.T. Grande Ospedale Metropolitano Niguarda, Milan, Italy; 11 Department of Clinical Biochemistry, Sultan Qaboos University Hospital, Muscat, Oman; 12 Weill Cornell Medicine Qatar, Doha, Qatar

## Abstract

The aim of this meta-analysis of randomized placebo-controlled clinical trials was to assess the effect of ezetimibe on plasma lipoprotein(a) concentrations. Only randomized placebo-controlled trials investigating the impact of ezetimibe treatment on cholesterol lowering that include lipoprotein(a) measurement were searched in PubMed-Medline, SCOPUS, Web of Science and Google Scholar databases (from inception to February 26^th^, 2018). A random-effects model and generic inverse variance method were used for quantitative data synthesis. Sensitivity analysis was conducted using the leave-one-out method. A weighted random-effects meta-regression was performed to evaluate the impact of potential confounders on lipoprotein concentrations. This meta-analysis of data from 10 randomized placebo-controlled clinical trials (15 treatment arms) involving a total of 5188 (3020 ezetimibe and 2168 control) subjects showed that ezetimibe therapy had no effect on altering plasma Lp(a) concentrations (WMD: −2.59%, 95% CI: −8.26, 3.08, *p* = 0.370; I^2^ = 88.71%, *p*_(Q)_ < 0.001). In the subgroup analysis, no significant alteration in plasma Lp(a) levels was observed either in trials assessing the impact of monotherapy with ezetimibe versus placebo (WMD: −4.64%, 95% CI: −11.53, 2.25, *p* = 0.187; I^2^ = 65.38%, *p*_(Q)_ = 0.005) or in trials evaluating the impact of adding ezetimibe to a statin versus statin therapy alone (WMD: −1.04%, 95% CI: −6.34, 4.26, *p* = 0.700; I^2^ = 58.51%, *p*_(Q)_ = 0.025). The results of this meta-analysis suggest that ezetimibe treatment either alone or in combination with a statin does not affect plasma lipoprotein(a) levels.

## Introduction

Ezetimibe inhibits absorption of cholesterol at the brush border of the small intestine via the sterol transporter, Niemann-Pick C1-Like1 (NPC1L1)^1^. This leads to a decreased delivery of cholesterol to the liver, reduction of hepatic cholesterol stores and an increased clearance of cholesterol from the blood and studies have shown a reduction in LDL-cholesterol (LDL-C) by 17% when used alone^2^ and a reduction of 14–25% in combination with statins^3^. The IMPROVE-IT (Improved Reduction of Outcomes: Vytorin Efficacy International Trial) in 18144 subjects with recent acute coronary syndrome showed that ezetimibe 10 mg added to simvastatin 40 mg daily reduced LDL-C by 16% with a 6% reduction in the risk of the primary cardiovascular outcome^4^.

Lipoprotein(a) (Lp(a)) is a low-density lipoprotein (LDL)-like particle with proatherogenic, prothrombotic and proinflammatory properties that make it a significant contributor to atherothrombotic events^5–10^. Lp(a) has been suggested to be an independent risk factor for cardiovascular disease with an increased odds ratio of 1.6^11^; however, a large meta-analysis of 36 prospective studies showed the risk ratio was 1.13^12^. Lp(a) is associated with acute coronary syndrome^13^ and increased levels are found commonly in patients with premature coronary heart disease^14^. Therefore, therapeutic approaches capable of reducing elevated plasma Lp(a) concentrations may be important.

Kinetic studies have concluded that Lp(a) levels are mainly regulated by differences in biosynthesis, and biosynthetic changes have been associated to the Lp(a) lowering activity of anacetrapib^15^. Recent reports have also indicated the role of plasminogen receptor KT in Lp(a) cell internalization, which is followed by recycling of the apo(a) component^16^. Additional to these mechanisms, there is evidence showing that Lp(a) serves as an acute-phase reactant and its biosynthesis is augmented by inflammation^17–19^. Ezetimibe is known to possess anti-inflammatory activity and may therefore affect Lp(a) production^20,23^. The involvement of the LDL receptor (LDLR) in the catabolism and clearance of plasma Lp(a) has also been discussed^24,25^. On the other hand, ezetimibe has been reported to potentiate the stimulating activity of statins on *LDLR* gene expression^26^. Given these lines of evidence, it might be plausible that the benefits of ezetimibe in improving outcomes, such as that observed in the IMPROVE-IT study, may in part be due to a decrease in Lp(a) levels and this meta-analysis of all randomized controlled trials was undertaken to determine if ezetimibe therapy does decrease Lp(a) levels.

## Methods

### Search Strategy

This study was designed according to the guidelines of the 2009 preferred reporting items for systematic reviews and meta-analysis (PRISMA) statement^27^. PubMed-Medline, SCOPUS, Web of Science and Google Scholar databases were searched using the following search terms within titles and abstracts (also in combination with MESH terms): (ezetimibe) AND (lipoprotein(a) OR “lipoprotein (a)” OR Lp(a) OR “Lp (a)”). Sensitivity of the search strategy was increased by using the wild-card term “*”. The search was limited to articles published in English language. The literature was searched from inception to February 26, 2018.

### Study Selection

Original studies were included if they met the following inclusion criteria: (i) being a randomized placebo-controlled trial with either parallel or cross-over design, (ii) investigating the impact of ezetimbe *vs*. placebo (or ezetimibe plus a statin *vs*. statin alone) on plasma/serum concentrations of Lp(a), and, (iii) presentation of sufficient information on Lp(a) concentrations at baseline and at the end of follow-up in each group or providing the net change values. Exclusion criteria were: (i) non-randomized controlled trials, (ii) lack of a control group for ezetimibe treatment, (iii) observational studies with case-control, cross-sectional or cohort design, and (iv) lack of sufficient information on baseline or follow-up Lp(a) concentrations.

### Data extraction

Eligible studies were reviewed and the following data were abstracted: (1) first author’s name; (2) year of publication; (3) country were the study was performed; (4) study design; (5) number of participants in the ezetimibe and control groups; (6) comedications including statins; (7) dose of ezetimibe; (8) treatment duration; (9) age, gender and body mass index (BMI) of study participants; (9) method of Lp(a) assay; and (10) data regarding baseline and follow-up plasma concentrations of Lp(a).

### Quality assessment

Included studies were systematically evaluated for the risk of bias according to the Cochrane criteria^28^, and as previously described, that included the assessment of each study for: adequacy of sequence generation, allocation concealment, blinding, addressing of dropouts (incomplete outcome data) and selective outcome reporting. According to the recommendations of the Cochrane Handbook, a judgment of “yes” indicated low risk of bias, while “no” indicated high risk of bias. Labeling an item as “unclear” indicated an unclear or unknown risk of bias.

### Quantitative Data Synthesis

Comprehensive Meta-Analysis (CMA) V2 software (Biostat, NJ)^29^ was used to undertake the meta-analysis. Effect size was calculated as: (measure at the end of follow-up in the treatment group – measure at baseline in the treatment group) – (measure at the end of follow-up in the control group – measure at baseline in the control group). A random-effects model (using DerSimonian-Laird method) and the generic inverse variance weighting method were used to compensate for the heterogeneity of studies in terms of study design, treatment duration, and the characteristics of populations being studied^30^. All values were collated as percentage changes. Standard deviations (SDs) of the mean difference were calculated using the following formula: SD = square root [(SD_pre-treatment_)^2^ + (SD_post-treatment_)^2^ − (2 R × SD_pre-treatment_ × SD_post-treatment_)], assuming a correlation coefficient (R) = 0.5^31–33^. Heterogeneity index was assessed using I^2^ index and Cochrane Q test. If the outcome measures were reported in median and range (or 95% confidence interval), mean and standard SD values were estimated using the method described by Wan *et al*.^34^. Where standard error of the mean (SEM) was only reported, SD was estimated using the following formula: SD = SEM × sqrt (*n*), where *n* is the number of subjects. Effect sizes were expressed as weighted mean difference (WMD) and 95% confidence interval (CI). If the outcome measures were reported in median and range (or 95% CI), mean and SD values were estimated using the method described by Wan *et al*.^34^. In order to evaluate the influence of each study on the overall effect size, a sensitivity analysis was conducted using the leave-one-out method (i.e., removing one study each time and repeating the analysis)^35–38^.

### Meta-regression

As potential confounder of treatment response, baseline plasma Lp(a) levels were entered into a random-effects meta-regression model to explore their association with the estimated effect size on plasma Lp(a) concentrations^37^.

### Publication bias

Evaluation of funnel plot, Begg’s rank correlation and Egger’s weighted regression tests were employed to assess the presence of publication bias in the meta-analysis^38^. When there was an evidence of funnel plot asymmetry, potentially missing studies were imputed using the “trim and fill” method^39^.

## Results

### Characteristics of included studies

**Figure 1:**
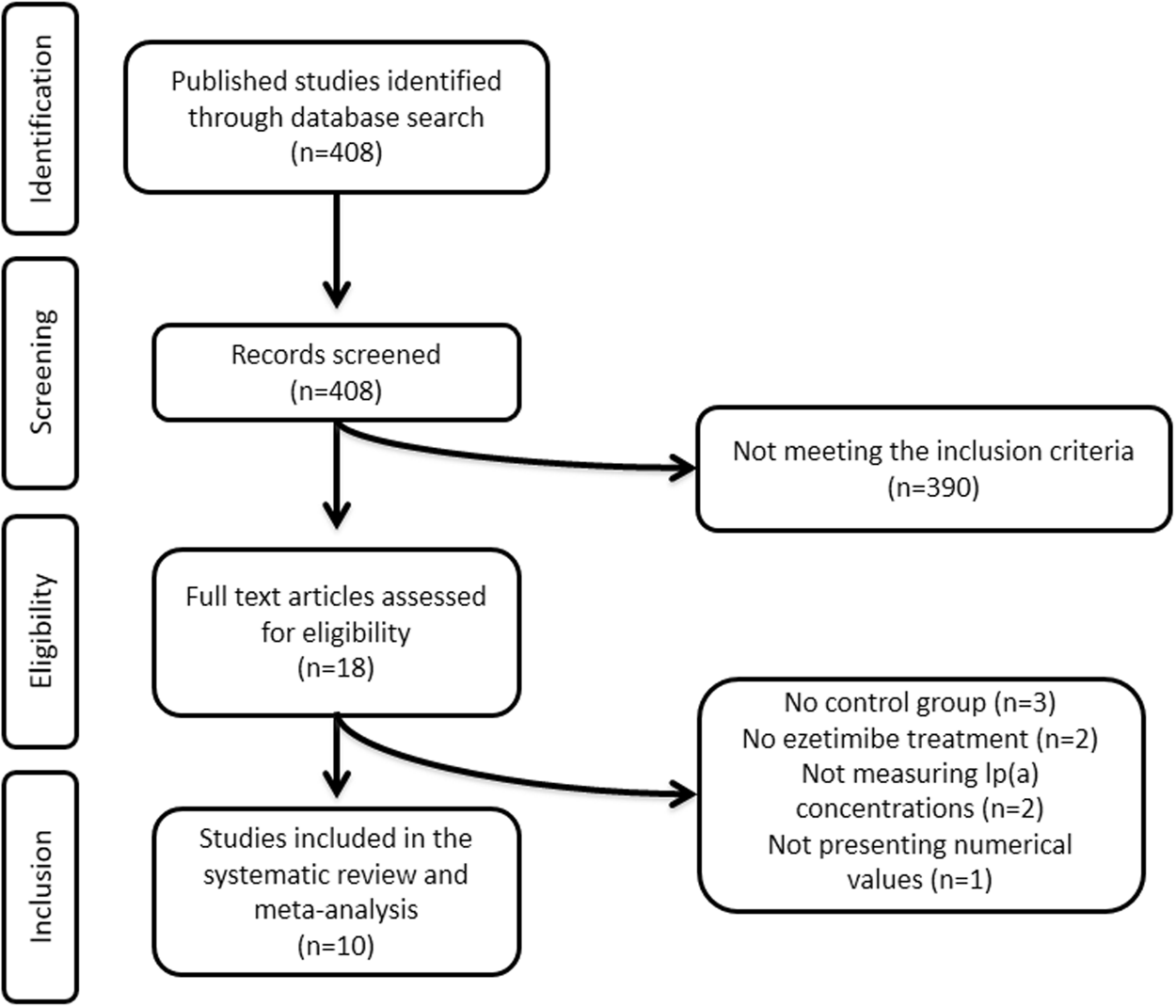
Flow chart that detailing the studies identified, screened, those that were eligible and those that were included into the meta-analysis.

A total of 408 clinical trials were identified of which 390 did not meet the inclusion criteria and were excluded. Eighteen full-text articles were carefully reviewed for eligibility from which 8 studies were excluded due to the lack of control group (n = 3), no ezetimibe arm (n = 2), lp(a) concentrations were not measured (n = 2), and not presenting numerical values (n = 1). After this assessment, 10 clinical trials were selected and included in this systematic review and meta-analysis. Flow chart that detailing the studies identified, screened, those that were eligible and those that were included into the meta-analysis is shown in Fig. 1.

Data were pooled from 10 clinical trials comprising a total of 5188 subjects, including 3020 and 2168 participants in the ezetimibe therapy and control arms (individuals of the cross-over trials were considered in treatment and control groups), respectively. Selected studies were published between 2002 and 2012. The range of intervention periods was from 5 weeks^40^ to 12 weeks^3,41–47^. Study designs of included trials were parallel^3,41–48^ and cross-over group^40^. Evaluated studies enrolled subjects with primary hypercholesterolemia^3,41–48^ and severe hypercholesterolemia^40^. Characteristics of the included clinical trials are shown in Table 1.

**Table 1:** Demographic characteristics of the included studies.

Author	Study design	Target Population	Treatment duration	n	Study groups	Age, years	Female (n, %)	Baseline BMI, (kg/m^2^)	Baseline Total cholesterol (mg/dl)	Baseline LDL cholesterol (mg/dl)	Baseline HDL cholesterol (mg/dl)	Baseline Triglycerides (mg/dl)	Baseline lipoprotein(a) (mg/dl)
Ballantyne *et al*.^43^	Randomized, double-blind, placebo-controlled	Primary hypercholeste-rolemia	12 weeks	60 65 248 255	Placebo Ezetimibe 10 mg/day Atorvastatin 10-80 mg/day Ezetimibe 10 mg/day + atorvastatin 10-80 mg/day	56.9 ± 12.1 56.7 ± 11.7 57.8 ± 11.7 58.7 ± 11.4	31 (52) 36 (55) 153 (62) 148 (58)	ND ND ND ND	261.8 ± 27.1 259.1 ± 28.2 268.8 ± 23.6 267.2 ± 23.9	177.9 ± 20.9 175.2 ± 21.7 179.8 ± 23.6 179.8 ± 23.9	50.3 ± 11.6 50.7 ± 12.0 53.8 ± 12.5 50.7 ± 12.7	141.7* 141.7* 150.6* 168.3*	ND ND ND ND
Davidson *et al*.^43^	Randomized, double-blind, placebo-controlled	Primary hypercholeste-rolemia	12 weeks	70 61 263 274	Placebo Ezetimibe 10 mg/day Simvastatin 10–80 mg/day Ezetimibe 10 mg/day + simvastatin 10–80 mg/day	58.8 (25–84)** 60.3 (35–84)** 56.4 (25–87)** 57.6 (27–83)**	39 (56) 37 (61) 153 (58) 148 (54)	ND ND ND ND	ND ND ND ND	177.4 ± 21.7 181.3 ± 23.0 178.5 ± 20.0 176.3 ± 19.9	52.3 ± 12.1 51.0 ± 11.5 51.0 ± 10.9 50.4 ± 12.2	170.9 ± 68.5 190.3 ± 68.2 168.7 ± 59.8 178.8 ± 65.1	30.1^†^ 34.7^†^ 33.0^†^ 30.8^†^
Dujovne *et al*.^43^	Randomized, double-blind, placebo-controlled	Primary hypercholeste-rolemia	12 weeks	226 666	Placebo Ezetimibe 10 mg/day	58.1 (30–85)** 57.9 (18–85)**	124 (55) 334 (50)	28.4 (19.4–49.5)** 28.6 (17.5–47)**	254.5^†^ 252.8^†^	168.0^†^ 167.8^†^	52.2^†^ 52.1^†^	174.8^†^ 169.0^†^	27.5^†^ 33.5^†^
Geiss *et al*.^43^	Randomized, double-blind, placebo-controlled, cross-over	Severe hypercholeste-rolemia	5 weeks	20 20 20	Overall Placebo Ezetimibe 10 mg/day	56 ± 9	10 (50)	27.5 ± 4.0	394.4^†^	317.5^†^	46.4^†^	158.5^†^	ND 32 ± 20 33 ± 27
Goldberg *et al*.^43^	Randomized, double-blind, placebo-controlled	Primary hypercholeste-rolemia	12 weeks	93 92 349 353	Placebo Ezetimibe 10 mg/day Simvastatin 10–80 mg/day Ezetimibe 10 mg/day + simvastatin 10–80 mg/day	ND ND ND ND	55 (59) 57 (62) 177 (51) 184 (52)	ND ND ND ND	258 ± 32 262 ± 30 259 ± 30 260 ± 30	174 ± 28 176 ± 26 175 ± 25 175 ± 27	50 ± 12 51 ± 13 49 ± 12 51 ± 13	162 ± 83^†^ 163 ± 104^†^ 167 ± 89^†^ 169 ± 93^†^	37 ± 38 35 ± 30 29 ± 27 31 ± 31
Kerzner *et al*.^43^	Randomized, double-blind, placebo-controlled	Primary hypercholeste-rolemia	12 weeks	64 72 220 192	Placebo Ezetimibe 10 mg/day Lovastatin 10–40 mg/day Ezetimibe 10 mg/day + lovastatin 10–40 mg/day	58 ± 12 55 ± 11 56 ± 12 57 ± 11	40 (63) 41 (57) 132 (60) 106 (55)	ND ND ND ND	266 ± 24 264 ± 25 265 ± 29 262 ± 27	178 ± 24 178 ± 16 178 ± 14 176 ± 13	54 ± 16 51 ± 8 51 ± 14 50 ± 13	168 ± 64 170 ± 59 178 ± 59 172 ± 55	34 ± 32 35 ± 33 35 ± 29 35 ± 27
Knopp *et al*.^43^	Randomized, double-blind, placebo-controlled	Primary hypercholeste-rolemia	12 weeks	205 622	Placebo Ezetimibe 10 mg/day	57.6 (24–79)** 58.3 (20–86)**	110 (54) 320 (51)	29.6 (19.4–45.7)** 29.1 (17.8–49.6)**	248.6^†^ 249.0^†^	164.3^†^ 165.1^†^	51.0^†^ 52.2^†^	170.9^†^ 163.0^†^	33.6^†^ 30.8^†^
Melani *et al*.^43^	Randomized, double-blind, placebo-controlled	Primary hypercholeste-rolemia	12 weeks	65 64 205 204	Placebo Ezetimibe 10 mg/day Pravastatin 10–40 mg/day Ezetimibe 10 mg/day + pravastatin 10–40 mg/day	53.4 (32–76)** 52.0 (26–75)** 55.1 (23–84)** 56.9 (20–86)**	34 (52) 41 (64) 104 (51) 121 (59)	ND ND ND ND	ND ND ND ND	177.9 ± 19.3 177.9 ± 23.2 177.9 ± 23.2 177.9 ± 19.3	50.3 ± 11.6 50.3 ± 11.6 50.3 ± 11.6 50.3 ± 11.6	159.4 ± 62.0 177.1 ± 62.0 177.1 ± 62.0 177.1 ± 62.0	33.6^†^ 30.8^†^ 30.8^†^ 30.8^†^
Moutzouri *et al*.^43^	Randomized, open-label, controlled	Primary hypercholeste-rolemia	12 weeks	30 30	Simvastatin 40 mg/day Ezetimibe 10 mg/day + simvastatin 40 mg/day	56.9 ± 13 56.9 ± 11	14 (46) 18 (60)	29 ± 5 29 ± 5	260 ± 45 271 ± 35	174 ± 41 179 ± 26	60 ± 12 61 ± 14	112 (58–129)‡ 119 (67–142) ‡	7.5 (2.3–21.3)‡ 4.7 (2.4–54.1)‡
Saougos *et al*.^43^	Open-label, controlled	Primary hypercholeste-rolemia	8 weeks	50 50	Ezetimibe 10 mg/day Rosuvastatin 10 mg/day	48.1 ± 19.5 54.6 ± 14.6	27 (54) 31 (62)	24.5 ± 7.9 25.8 ± 4.2	263 ± 34 297 ± 50	170 ± 30 208 ± 42	61 ± 15 58 ± 11	141 ± 44 141 ± 53	4.2 (2.0–6.1)‡ 4.0 (2.0–7.4)‡

Values are expressed as mean ± SD.

*Median.

**Mean (IQR).

^†^Mean.

^‡^Median (IQR).

Abbreviations: ND, no data; BMI, body mass index; IQR, interquartile range.

Demographic characteristics of the studies that were included in this meta-analysis.

### Lp(a) assay methods

The majority of the studies included in the meta-analysis quantified Lp(a) levels by competitive enzyme-linked immunosorbent assay^3,41–46,48^, while other trials determined Lp(a) levels by a nephelometric assay (Dade Behring)^40,47^.

### Risk of bias in the included studies

Several studies showed low risk of bias regarding random sequence generation, only one trial had high risk of bias for this parameter^48^. Most of the included studies had a lack of information about the allocation concealment, and a high risk of bias was found in one trial^48^. Several studies presented insufficient information on the blinding process of the participants, study personnel and the outcome assessors, and two trials showed high risk of bias^47,48^. Almost all of the assessed studies showed that there was a low risk of bias for incomplete outcome data, only one trial exhibited limited information^41^. Finally, all selected studies presented low risk of bias regarding selective outcome reporting. Cochrane guidelines applied to the assessment of quality of bias in the studies that were included in the meta-analysis are shown in Table 2.

**Table 2:** Quality of bias assessment of the included studies according to the Cochrane guidelines.

Study	Sequence generation	Allocation concealment	Blinding of participants, personnel and outcome assessors	Incomplete outcome data	Selective outcome reporting	Other sources of bias
Ballantyne *et al*.^43^	U	U	U	U	L	U
Davidson *et al*.^43^	L	U	U	L	L	U
Dojovne *et al*.^43^	L	L	U	L	L	U
Geiss *et al*.^43^	U	U	U	L	L	U
Goldberg *et al*.^43^	L	U	U	L	L	U
Kerzner *et al*.^43^	U	U	U	L	L	U
Knopp *et al*.^43^	L	U	U	L	L	U
Melani *et al*.^43^	L	U	U	L	L	U
Moutzouri *et al*.^43^	L	U	H	L	L	U
Saougos *et al*.^43^	H	H	H	L	L	U

L, low risk of bias; H, high risk of bias; U, unclear risk of bias.

Cochrane guidelines applied to the assessment of quality of bias in the studies that were included in the meta-analysis.

### Effect of Ezetimibe on plasma Lp(a) concentrations

**Figure 2:**
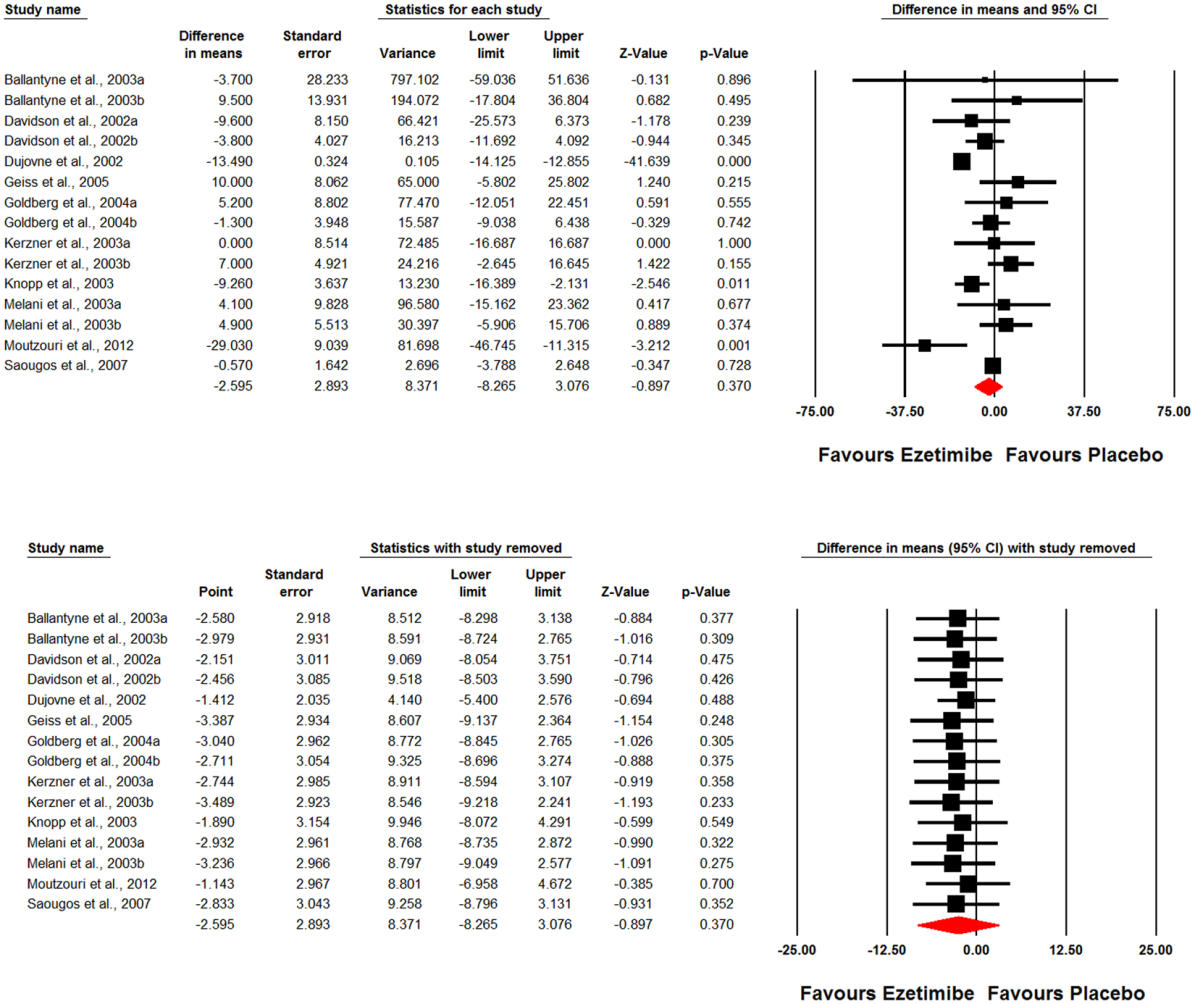
Weighted mean difference and 95% confidence intervals for the impact of ezetimibe on plasma Lp(a) concentrations displayed as a Forest plot. The results of leave-one-out sensitivity analysis is shown in the lower panel.

Overall, 10 studies comprising 15 treatment arms were included in the meta-analysis. Meta-analysis did not suggest a significant change in plasma Lp(a) concentrations following treatment with ezetimibe (WMD: −2.59%, 95% CI: −8.26, 3.08, *p* = 0.370; I^2^ = 88.71%, *p*_(Q)_ < 0.001) (Fig. 2). The effect size was robust in sensitivity analysis (Fig. 2) and the effect size remained significant or marginally significant after omission of each single study from the meta-analysis.

**Figure 3:**
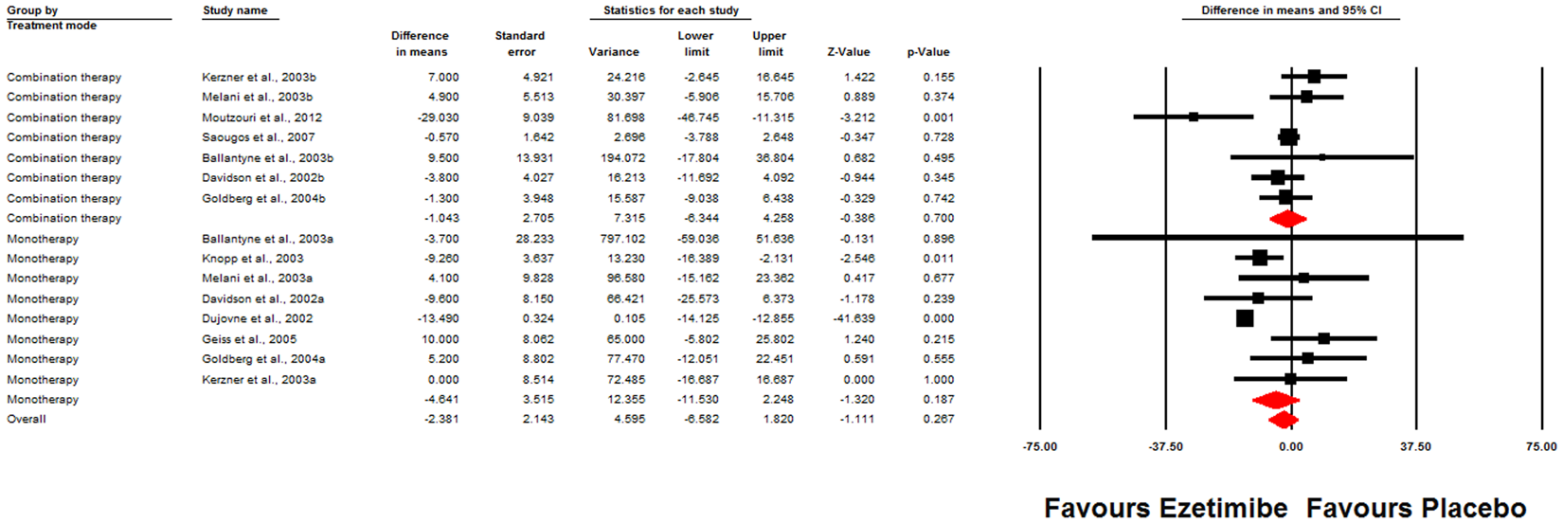
Weighted mean difference and 95% confidence intervals for the impact of ezetimibe on plasma Lp(a) concentrations in subgroups of trials administering ezetimibe as monotherapy or in combination with statins displayed as a Forest plot. The results of leave-one-out sensitivity analysis is shown in the lower panel.

In the subgroup analysis, no significant alteration in plasma Lp(a) levels was observed either in trials assessing the impact of monotherapy with ezetimibe versus placebo (WMD: −4.64%, 95% CI: −11.53, 2.25, *p* = 0.187; I^2^ = 65.38%, *p*_(Q)_ = 0.005) or in trials evaluating the impact of adding ezetimibe to a statin versus statin therapy alone (WMD: −1.04%, 95% CI: −6.34, 4.26, *p* = 0.700; I^2^ = 58.51%, *p*_(Q)_ = 0.025) (Fig. 3).

### Meta-regression

**Figure 4:**
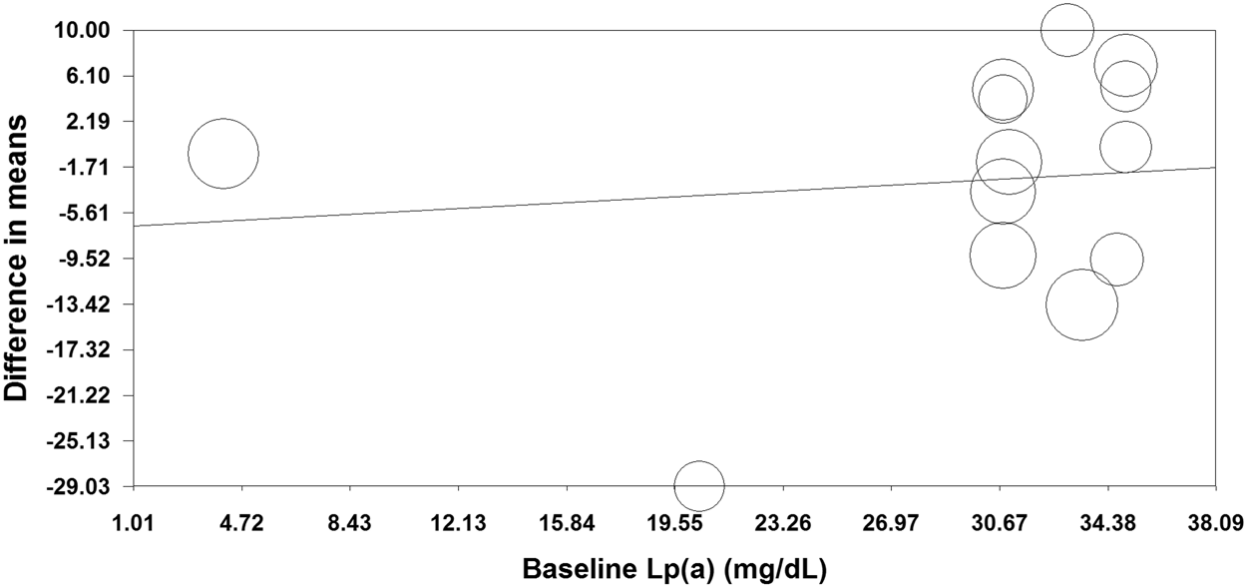
Meta-regression bubble plot of the association between mean changes in plasma Lp(a) concentrations following ezetimibe treatment with baseline plasma Lp(a) concentrations. The size of each circle is inversely proportional to the variance of change.

To assess the impact of baseline Lp(a) concentrations on the effect of ezetimibe on plasma Lp(a) levels, a random-effects meta-regression was undertaken. The result did not suggest a significant association under a random-effects meta-regression model (slope: 0.135; 95% CI: −0.504, 0.774; *p* = 0.679) (Fig. 4).

### Publication bias

**Figure 5:**
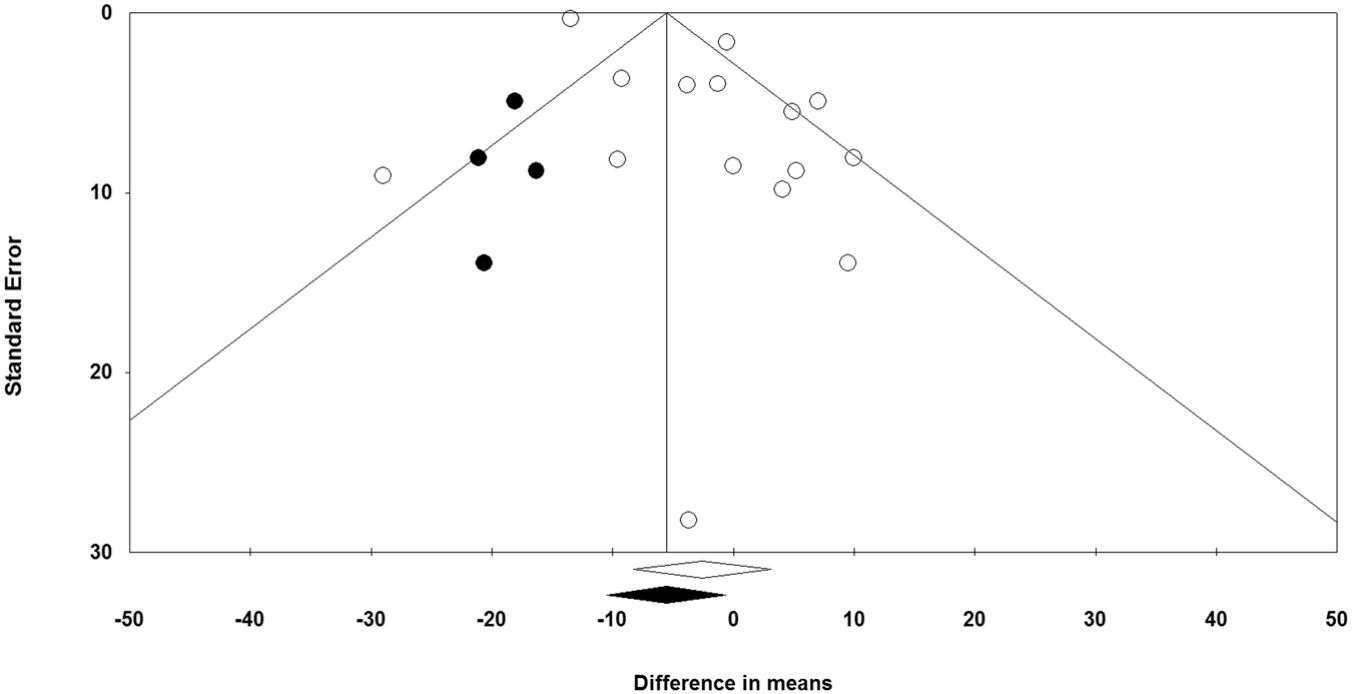
Publication bias in the studies reporting the impact of ezetimibe treatment on plasma Lp(a) concentrations displayed as a Funnel plot. Open and closed circles represent reported studies and potentially missing studies imputed using “trim and fill” method.

Visual inspection of Begg’s funnel plots showed an asymmetry in the meta-analyses of ezetimibe’s effects on plasma Lp(a) concentrations. This asymmetry was corrected by imputing four potentially missing studies using “trim and fill” method, yielding a corrected effect size of −5.56 (95% CI: −10.50, −0.61) (Fig. 5). Consistently, Egger’s regression test (*t = *3.38, df = 13, *p* = 0.005) did suggest the presence of publication bias. However, there was no sign of publication bias according to the Begg’s rank correlation test (tau = −0.27, *z* = 1.39, *p* = 0.166).

## Discussion

In this meta-analysis of randomized placebo-controlled clinical trials, ezetimibe therapy was not related to a significant reduction of plasma Lp(a) levels. This result was robust in the sensitivity analysis and was confirmed both when ezetimibe was used as monotherapy and in combination with a statin. In a previous meta-analysis, ezetimibe monotherapy was found to cause a significant but small and not clinically relevant reduction (7%) in plasma Lp(a) levels^49^. The present meta-analysis provides a more robust confirmation on the lack of any Lp(a)-lowering effect of ezetimibe compared with referenced meta-analysis because of including a larger number of randomized controlled trials (*n* = 10 *vs*. 7). Moreover, in addition to the impact of monotherapy, this meta-analysis also evaluated the impact of the combination of ezetimibe and statins (*vs*. statin monotherapy) on plasma Lp(a) levels that has not been the subject of any prior meta-analysis.

Lp(a) is associated with acute coronary syndrome^13^ and increased levels are found commonly in patients with premature coronary heart disease^14^. Other studies including Lp(a) in a predictive model for cardiovascular prevention have suggested that the inclusion of Lp(a) in the models does increase their predictive value^50–53^. However, whilst Lp(a) elevation is associated with cardiovascular disease it is still unclear if there is a benefit in therapeutically targeting its reduction specifically. Although no specific therapy for lowering Lp(a) is as yet approved, the body of evidence supports the reducing effects by lipoprotein apheresis^54^, proprotein convertase subtilisin/kexin type 9 (PCSK9) inhibitors^55^, estrogen mimics and selective estrogen receptor modulators^56–58^, nicotinic acid^59^ and some nutraceuticals^60–63^. Moreover, selective apo(a) antisense therapy has shown promising lowering effects in patients with elevated Lp(a) in phase II trials^64^. With this background, an additional pharmacological tool for Lp(a)-lowering would be desirable.

Ezetimibe has a modest LDL-C-lowering efficacy that has been associated with a significant improvement of cardiovascular prognosis in the IMPROVE-IT trial^4^. Given the reduction in plasma LDL-C levels with ezetimibe and the similarity in the lipid composition between Lp(a) and LDL particles^2,3^, it might be hypothesized that ezetimibe would have also an impact on plasma Lp(a) levels. However, this meta-analysis collecting the inconsistent results of 10 randomized placebo-controlled trials was convincing that this is not the case, despite few studies reported a beneficial effect of ezetimibe on plasma Lp(a) levels^3,42,45,47^. Clear reasons explaining the divergent results of the included trials did not emerge from the analytical comparison of each of them; however, we cannot rule out the hypothesis that untested or unreported data might have contributed to such an inconsistency. Thus, for instance, whether baseline BMI might have affected the Lp(a)-lowering efficacy of ezetimibe cannot be excluded; accordingly, in 3^3,42,45,47^ out of 4 trials^3,42,45,47^ reporting a significant impact of ezetimibe on Lp(a), average BMI was almost 29, whereas it was 26 in the only 2 trials^40,48^ reporting the baseline BMI value of the study participants and which also showed a neutral effect of ezetimibe on Lp(a). Hence, further trials with complete reporting of the baseline characteristics of the study subjects are warranted.

This meta-analysis has a number of limitations. Firstly, effects of ezetimibe therapy on Lp(a) were not the primary aim of the clinical trials and the studies were not powered for this. Secondly, there were only 10 trials available to be analysed giving a modest though robust number of subjects to undertake the analysis. Thirdly, the longest duration of the trials exploring the Lp(a)-lowering effect of ezetimibe was 12 weeks, which might be too short to allow a greater impact on plasma Lp(a) levels. Finally, given the skewed distribution of Lp(a) values in the population, further clarification would benefit from pooled analyses of individual patient data.

## Conclusion

The results of this meta-analysis suggest that ezetimibe treatment does not reduce plasma lipoprotein(a) levels and therefore Lp(a) reduction does not contribute to its therapeutic effect in cardiovascular prevention.

## References

[1] Sudhop T (2002). Inhibition of intestinal cholesterol absorption by ezetimibe in humans. Circulation.

[2] Knopp RH (2003). Effects of ezetimibe, a new cholesterol absorption inhibitor, on plasma lipids in patients with primary hypercholesterolemia. European heart journal.

[3] Davidson MH (2002). Ezetimibe coadministered with simvastatin in patients with primary hypercholesterolemia. Journal of the American College of Cardiology.

[4] Cannon CP (2015). Ezetimibe Added to Statin Therapy after Acute Coronary Syndromes. The New England journal of medicine.

[5] Ellis KL, Boffa MB, Sahebkar A, Koschinsky ML, Watts GF (2017). The renaissance of lipoprotein(a): Brave new world for preventive cardiology?. Progress in Lipid Research.

[6] Ferretti, G. *et al*. Lipoprotein(a): A missing culprit in the management of athero-thrombosis? *Journal of Cellular Physiology*, 10.1002/jcp.26050 (2017). 10.1002/jcp.26050 28608522

[7] Pirro M (2017). Lipoprotein(a) and inflammation: A dangerous duet leading to endothelial loss of integrity. Pharmacological Research.

[8] Kronenberg F, Utermann G (2013). Lipoprotein(a): resurrected by genetics. J Intern Med.

[9] Schmidt K, Noureen A, Kronenberg F, Utermann G (2016). Structure, function, and genetics of lipoprotein (a). J Lipid Res.

[10] Seed M (1990). Relation of serum lipoprotein(a) concentration and apolipoprotein(a) phenotype to coronary heart disease in patients with familial hypercholesterolemia. The New England journal of medicine.

[11] Bennet A (2008). Lipoprotein(a) levels and risk of future coronary heart disease: large-scale prospective data. Archives of internal medicine.

[12] Erqou S (2009). Lipoprotein(a) concentration and the risk of coronary heart disease, stroke, and nonvascular mortality. Jama.

[13] Dangas, G. *et al*. Correlation of serum lipoprotein(a) with the angiographic and clinical presentation of coronary artery disease. *The American journal of cardiology* 83, 583–585, a587 (1999). 10.1016/s0002-9149(98)00917-5 10073865

[14] Genest JJ (1992). Familial lipoprotein disorders in patients with premature coronary artery disease. Circulation.

[15] Thomas T (2017). CETP (Cholesteryl Ester Transfer Protein) Inhibition With Anacetrapib Decreases Production of Lipoprotein(a) in Mildly Hypercholesterolemic Subjects. Arteriosclerosis, thrombosis, and vascular biology.

[16] Sharma M, Redpath GM, Williams MJ, McCormick SP (2017). Recycling of Apolipoprotein(a) After PlgRKT-Mediated Endocytosis of Lipoprotein(a). Circulation research.

[17] Noma A (1994). Lp(a): an acute-phase reactant?. Chem Phys Lipids.

[18] Wade DP (1993). 5′ control regions of the apolipoprotein(a) gene and members of the related plasminogen gene family. Proc Natl Acad Sci USA.

[19] Wang J, Hu B, Kong L, Cai H, Zhang C (2008). Native, oxidized lipoprotein(a) and lipoprotein(a) immune complex in patients with active and inactive rheumatoid arthritis: plasma concentrations and relationship to inflammation. Clin Chim Acta.

[20] Tobaru T, Seki A, Asano R, Sumiyoshi T, Hagiwara N (2013). Lipid-lowering and anti-inflammatory effect of ezetimibe in hyperlipidemic patients with coronary artery disease. Heart Vessels.

[21] Tie C (2015). Ezetimibe Attenuates Atherosclerosis Associated with Lipid Reduction and Inflammation Inhibition. PLoS One.

[22] Qin L (2014). Anti-inflammatory activity of ezetimibe by regulating NF-kappaB/MAPK pathway in THP-1 macrophages. Pharmacology.

[23] Dolezelova E (2017). Effect of ezetimibe on plasma adipokines: a systematic review and meta-analysis. Br J Clin Pharmacol.

[24] Lambert G (2017). The complexity of lipoprotein (a) lowering by PCSK9 monoclonal antibodies. Clinical science.

[25] Watts, G. F. *et al*. Controlled study of the effect of proprotein convertase subtilisin-kexin type 9 inhibition with evolocumab on lipoprotein(a) particle kinetics. *European heart journal*, 10.1093/eurheartj/ehy122 (2018). 10.1093/eurheartj/ehy122 29566128

[26] Gouni-Berthold I (2008). Effects of ezetimibe and/or simvastatin on LDL receptor protein expression and on LDL receptor and HMG-CoA reductase gene expression: a randomized trial in healthy men. Atherosclerosis.

[27] Moher, D., L., A., Tetzlaff, J., Altman, D. G. & PRISMA Group. Preferred reporting items for systematic reviews and meta-analyses: the PRISMA statement. *BMJ* **339**, b2535, 10.1136/bmj.b2535 (2009). PMC3090117 21603045

[28] Higgins JPT, G. S. *Handbook for Systematic Reviews of Interventions*, Version 5.0.2 edn., (2009).

[29] Borenstein M. H. L., Higgins JPT. *Comprehensive Meta-analysis*. (2005).

[30] Sutton, A. J., A. K. Jones, D. R., Sheldon, T. A.,. Song F. *Methods for meta-analysis in medical research*. (2000).

[31] Sahebkar A (2015). Statin therapy reduces plasma endothelin-1 concentrations: A meta-analysis of 15 randomized controlled trials. Atherosclerosis.

[32] Sahebkar A (2015). Association between statin use and plasma D-dimer levels. A systematic review and meta-analysis of randomised controlled trials. Thromb Haemost.

[33] Sahebkar A (2015). Lack of efficacy of resveratrol on C-reactive protein and selected cardiovascular risk factors–Results from a systematic review and meta-analysis of randomized controlled trials. Int J Cardiol.

[34] Wan XWW, Liu J, Tong T (2014). Estimating Estimating the sample mean and standard deviation from the sample size, median, range and/or interquartile range. BMC Med Res Methodol..

[35] Sahebkar A (2014). A systematic review and meta-analysis of randomized controlled trials investigating the effects of curcumin on blood lipid levels. Clinical Nutrition.

[36] Sahebkar A, Cicero AFG, Simental-Mendía LE, Aggarwal BB, Gupta SC (2016). Curcumin downregulates human tumor necrosis factor-α levels: A systematic review and meta-analysis ofrandomized controlled trials. Pharmacological Research.

[37] Serban, C. *et al*. A systematic review and meta-analysis of the effect of statins on plasma asymmetric dimethylarginine concentrations. *Scientific Reports* **5**, 10.1038/srep09902 (2015). 10.1038/srep09902 PMC4429557 25970700

[38] Sahebkar A (2013). Does PPARgamma2 gene Pro12Ala polymorphism affect nonalcoholic fatty liver disease risk? Evidence from a meta-analysis. DNA Cell Biol.

[39] Duval S (2000). T. R. Trim and fill: A simple funnel-plot-based method of testing and adjusting for publication bias in meta-analysis. Biometrics.

[40] Geiss HC, Otto C, Hund-Wissner E, Parhofer KG (2005). K.G. Effects of ezetimibe on plasma lipoproteins in severely hypercholesterolemic patients treated with regular LDL-apheresis and statins. Atherosclerosis.

[41] Ballantyne CM (2003). Ezetimibe Study Group. Effect of ezetimibe coadministered with atorvastatin in 628 patients with primary hypercholesterolemia: a prospective, randomized, double-blind trial. Circulation.

[42] Dujovne CA (2002). Study Group. Efficacy and safety of a potent new selective cholesterol absorption inhibitor, ezetimibe, in patients with primary hypercholesterolemia. Am J Cardiol..

[43] Goldberg AC, S. A, Liu J, Capece R, Mitche YB, Ezetimibe Study Group. (2004). Efficacy and safety of ezetimibe coadministered with simvastatin in patients with primary hypercholesterolemia: a randomized, double-blind, placebo-controlled trial. Mayo Clin Proc..

[44] Kerzner B (2003). Ezetimibe Study Group. Efficacy and safety of ezetimibe coadministered with lovastatin in primary hypercholesterolemia. Am J Cardiol..

[45] Knopp RH (2003). Ezetimibe Study Group. Effects of ezetimibe, a new cholesterol absorption inhibitor, on plasma lipids in patients with primary hypercholesterolemia. Eur Heart J..

[46] Melani L (2003). Ezetimibe Study Group. Efficacy and safety of ezetimibe coadministered with pravastatin in patients with primary hypercholesterolemia: a prospective, randomized, double-blind trial. Eur Heart J..

[47] Moutzouri E (2012). Effect of simvastatin or its combination with ezetimibe on Toll-like receptor expression and lipopolysaccharide - induced cytokine production in monocytes of hypercholesterolemic patients. Atherosclerosis..

[48] Saougos VG (2007). AD. Differential effect of hypolipidemic drugs on lipoprotein-associated phospholipase A2. Arterioscler Thromb Vasc Biol..

[49] Awad K (2018). Effect of Ezetimibe Monotherapy on Plasma Lipoprotein(a) Concentrations in Patients with Primary Hypercholesterolemia: A Systematic Review and Meta-Analysis of Randomized Controlled Trials. Drugs.

[50] Willeit P (2014). Discrimination and net reclassification of cardiovascular risk with lipoprotein(a): prospective 15-year outcomes in the Bruneck Study. Journal of the American College of Cardiology.

[51] Kamstrup PR, Tybjaerg-Hansen A, Nordestgaard BG (2013). Extreme lipoprotein(a) levels and improved cardiovascular risk prediction. Journal of the American College of Cardiology.

[52] Kostner KM, Kostner GM, Wierzbicki AS (2018). Is Lp(a) ready for prime time use in the clinic? A pros-and-cons debate. Atherosclerosis.

[53] Kostner KM, Marz W, Kostner GM (2013). When should we measure lipoprotein (a)?. Eur Heart J.

[54] Ezhov MV (2015). Specific Lipoprotein(a) apheresis attenuates progression of carotid intima-media thickness in coronary heart disease patients with high lipoprotein(a) levels. Atheroscler Suppl.

[55] Karatasakis, A. *et al*. Effect of PCSK9 Inhibitors on Clinical Outcomes in Patients With Hypercholesterolemia: A Meta-Analysis of 35 Randomized Controlled Trials. *J Am Heart Assoc* **6**, 10.1161/JAHA.117.006910 (2017). 10.1161/JAHA.117.006910 PMC5779013 29223954

[56] Ferretti G (2017). Raloxifene Lowers Plasma Lipoprotein(a) Concentrations: a Systematic Review and Meta-analysis of Randomized Placebo-Controlled Trials. Cardiovascular Drugs and Therapy.

[57] Kotani K (2015). Tibolone decreases Lipoprotein(a) levels in postmenopausal women: A systematic review and meta-analysis of 12 studies with 1009 patients. Atherosclerosis.

[58] Sahebkar A (2017). The Effects of Tamoxifen on Plasma Lipoprotein(a) Concentrations: Systematic Review and Meta-Analysis. Drugs.

[59] Sahebkar A, Reiner Ž, Simental-Mendïa LE, Ferretti G, Cicero AFG (2016). Effect of extended-release niacin on plasma lipoprotein(a) levels: A systematic review and meta-analysis of randomized placebo-controlled trials. Metabolism: Clinical and Experimental.

[60] Panahi Y, Khalili N, Hosseini MS, Abbasinazari M, Sahebkar A (2014). Lipid-modifying effects of adjunctive therapy with curcuminoids-piperine combination in patients with metabolic syndrome: Results of a randomized controlled trial. Complementary Therapies in Medicine.

[61] Panahi Y (2017). Curcuminoids modify lipid profile in type 2 diabetes mellitus: A randomized controlled trial. Complementary Therapies in Medicine.

[62] Sahebkar A, Simental-Mendía LE, Stefanutti C, Pirro M (2016). Supplementation with coenzyme Q10 reduces plasma lipoprotein(a) concentrations but not other lipid indices: A systematic review and meta-analysis. Pharmacological Research.

[63] Serban, M. C. *et al*. Impact of L-carnitine on plasma lipoprotein(a) concentrations: A systematic review and meta-analysis of randomized controlled trials. *Scientific Reports* **6**, 10.1038/srep19188 (2016). 10.1038/srep19188 PMC4709689 26754058

[64] Viney NJ (2016). Antisense oligonucleotides targeting apolipoprotein(a) in people with raised lipoprotein(a): two randomised, double-blind, placebo-controlled, dose-ranging trials. Lancet.

