# Author Correction: Impact of ezetimibe on plasma lipoprotein(a) concentrations as monotherapy or in combination with statins: a systematic review and meta-analysis of randomized controlled trials

**DOI:** 10.1038/s41598-020-59903-6

**Published:** 2020-02-14

**Authors:** Amirhossein Sahebkar, Luis E. Simental-Mendía, Matteo Pirro, Maciej Banach, Gerald F. Watts, Cesare Sirtori, Khalid Al-Rasadi, Stephen L. Atkin

**Affiliations:** 1 Halal Research Center of IRI, FDA, Tehran, Iran; 2 Biotechnology Research Center, Pharmaceutical Technology Institute, Mashhad University of Medical Sciences, Mashhad, Iran; 3 Neurogenic Inflammation Research Center, Mashhad University of Medical Sciences, Mashhad, Iran; 4 School of Pharmacy, Mashhad University of Medical Sciences, Mashhad, Iran; 5 Biomedical Research Unit, Mexican Social Security Institute, Durango, Mexico; 6 Unit of Internal Medicine, Angiology and Arteriosclerosis Diseases, Department of Medicine, University of Perugia, Perugia, Italy; 7 Department of Hypertension, WAM University Hospital in Lodz, Medical University of Lodz, Zeromskiego 113, Lodz, Poland; 8 Polish Mother’s Memorial Hospital Research Institute (PMMHRI), Lodz, Poland; 9 School of Medicine, Faculty of Health and Medical Sciences, University of Western Australia, Perth, Australia; 10 Lipid Disorders Clinic, Cardiometabolic Services, Department of Cardiology, Royal Perth Hospital, GPO Box, X2213 Perth, Australia; 11 Centro Dislipidemie, A.S.S.T. Grande Ospedale Metropolitano Niguarda, Milan, Italy; 12 Department of Clinical Biochemistry, Sultan Qaboos University Hospital, Muscat, Oman; 13 Weill Cornell Medicine Qatar, Doha, Qatar

Correction to: *Scientific Reports* 10.1038/s41598-018-36204-7, published online 14 December 2018

The original version of this Article contained a typographical error in the spelling of the author Cesare Sirtori, which was incorrectly given as Cesare Sirotri.

Additionally, the original version of this Article omitted an affiliation for Amirhossein Sahebkar. The correct affiliations for Amirhossein Sahebkar are listed below:

Halal Research Center of IRI, FDA, Tehran, Iran.

Biotechnology Research Center, Pharmaceutical Technology Institute, Mashhad University of Medical Sciences, Mashhad, Iran.

Neurogenic Inflammation Research Center, Mashhad University of Medical Sciences, Mashhad, Iran.

School of Pharmacy, Mashhad University of Medical Sciences, Mashhad, Iran.

This has now been corrected in the HTML and PDF versions of this Article.

